# Money Based Reform and Distorted Doctor-patient Interaction: A Critique of the Recent Health Sector Evolution Plan in Iran

**Published:** 2017-04

**Authors:** Ahmad KALATEH SADATI

**Affiliations:** Dept. of Sociology, Faculty of Social Sciences, Yazd University, Yazd, Iran

## Dear Editor-in-Chief

Health system in Iran has several chronic issues. Alongside ineffective infrastructures, there are several problems such as; urbanization, change in lifestyle, prevalence of non-communicable diseases (NCDs) and HIV, as well as increase in a number of senior citizens (1). Despite these basic problems, the health system has another chronic problem that is the absence of any master plan. In addition, annually about 700 thousand Iranian people fall below the poverty line due to high cost of medicine.

Due to these issues recently, Health Sector Evolution Plan (HSEP) was hastily designed in 2014 based on money based planning. In order to reduce patients out of pocket (OOP) payment government hospitals were giving the instruction only to charge patients 10% of the total hospital cost (2). The idea was that physicians in governmental hospitals should be paid more based on the tariff booklet.

Although the main goal of the reform, which was reducing the OOP of population, was reached apparently, (we do not have rich evidence for this claim) there are several critiques. These include sustainability of the programs and equity of financing (2) and heavy financial burdens on government, neglect of primary health care, inefficient payment methods, scarce financial sources, unequal distribution of specialists, and disregarding outpatients in public sectors and patients in private hospitals (3). The reform should be criticized from different view called *Doctor-Patient Interaction (DPI).*

DPI is an important subject in sociology of medicine specifically in functionalist and critical views (4). While functionalism defines ‘sick role’ as the main duty of patient in communication with doctor (5) critical views criticize asymmetrical power relationships between two parties (6).

Among critical views, communicative action was defined versus strategic one. While communicative action “seek to reach an understanding about the action situation and their plans of action in order to coordinate their actions by way of agreement; strategic action involves the instrumentalization of social cooperation, strategic action is coordination of action by means of “influence”, where influence means the employment of inducements other than reasons (7). In this view, money is the medium of distorted interactions (6, 7). For example, a recent study showed that standards of medicine are the main reason for patients’ clinical dependency as a strategy of political economy (4). Here, political economy of clinic defines some general standards for maintain the dependency of patient to clinic as a general strategy.

In spite of limitation of studies in Iran, DPI leads to making patient clinical dependency (4), suppression the patients, dissatisfaction of patient and accompanies (4, 8, 9, 10).

Now, the main question is ‘what are the effects of HSEP on DPI in Iran’. Based on mentioned theoretical view HSEP led to speed up commercialization of medicine and DPI is defined by domination of money and its functions. Thus, medical treatment, as well as management, is governed by money. In this situation, doctor and patient do not reach a mutual understanding but they stand in asymmetrical power relationships, which lead to several problems as in addition to distorted DPI.

Finally, the reform increased level of inequality in society. Implementation of tariff booklet is the main cause in creating inequality and formation of class society. Tariff booklet has increased the payment of clinical physicians in Iran several times more in comparison to pre-plan. This increase in salary not only is unfair amongst the medical care groups but it also has created segregated society. Opponents claimed that in this reform some specialists earn as much as 100000 USD per month. While, workers salary was determined about 200 USD per month. aThus, the reform led to speed up formation of class society. According to social inequalities as the main part of social determinant of health (SDHs), thus HSEP not only cannot decline the SDHs effects but it can lead to several unpredicted public health problems due to increasing of social inequalities and formation of class distinctions in the society.
